# MetaCluster 5.0: a two-round binning approach for metagenomic data for low-abundance species in a noisy sample

**DOI:** 10.1093/bioinformatics/bts397

**Published:** 2012-09-03

**Authors:** Yi Wang, Henry C.M. Leung, S.M. Yiu, Francis Y.L. Chin

**Affiliations:** Department of Computer Science, The University of Hong Kong, Hong Kong

## Abstract

**Motivation:** Metagenomic binning remains an important topic in metagenomic analysis. Existing unsupervised binning methods for next-generation sequencing (NGS) reads do not perform well on (i) samples with low-abundance species or (ii) samples (even with high abundance) when there are many extremely low-abundance species. These two problems are common for real metagenomic datasets. Binning methods that can solve these problems are desirable.

**Results:** We proposed a two-round binning method (MetaCluster 5.0) that aims at identifying both low-abundance and high-abundance species in the presence of a large amount of noise due to many extremely low-abundance species. In summary, MetaCluster 5.0 uses a filtering strategy to remove noise from the extremely low-abundance species. It separate reads of high-abundance species from those of low-abundance species in two different rounds. To overcome the issue of low coverage for low-abundance species, multiple *w* values are used to group reads with overlapping *w*-mers, whereas reads from high-abundance species are grouped with high confidence based on a large *w* and then binning expands to low-abundance species using a relaxed (shorter) *w*. Compared to the recent tools, TOSS and MetaCluster 4.0, MetaCluster 5.0 can find more species (especially those with low abundance of say 6× to 10×) and can achieve better sensitivity and specificity using less memory and running time.

**Availability:** http://i.cs.hku.hk/~alse/MetaCluster/

**Contact:** chin@cs.hku.hk

## 1 INTRODUCTION

Metagenomics is the study of genomes of multiple species from environmental samples, such as soil, sea water and the human gut. Successful metagenomic projects provide deeper insight into the microbial world. For example, the diversity of microbes in the human gut has been found to be related to common diseases such as inflammatory bowel disease (IBD) (Qin *et al.*, 2010) and gastrointestinal disturbance (Khachatryan *et al.*, 2008). High-throughput next-generation sequencing (NGS) techniques can sequence reads (short DNA fragments) from a sample containing genomes of multiple species. An important step in metagenomic analysis is grouping reads from similar species together, which is known as *binning*.

Existing binning algorithms fall into two main categories, supervised methods and unsupervised methods. Supervised methods (Brady and Salzberg, 2009; McHardy *et al.*, 2006) align reads to known genomes and group reads aligned to similar genomes together. Since up to 99% (Eisen, 2007) of bacteria found in environmental samples are unknown or cannot be cultured and separated in laboratories (Amann *et al.*, 1990), most reads cannot be aligned and binned. Instead of aligning reads to known genomes directly, some semi-supervised methods use taxonomic markers [e.g. recA, rpoB and 16S rRNA (Cole *et al.*, 2005)] to classify reads into different groups. However, the precision of these methods may be low because species may contain multiple markers and different species may share markers (Case *et al.*, 2007). Moreover, since only a small part (*<*1%) of the genome (or reads) contains these taxonomic markers (Garcia Martin *et al.*, 2008), most of the reads cannot be binned by these methods.

When the corresponding genomes are unknown, unsupervised methods usually bin reads based on three observations: (A) the *k*-mer frequency from reads of a genome is usually linearly proportional to that of the genome's abundance (Wu and Ye, 2011); (B) sufficiently long *w*-mers are usually unique in each genome (Fofanov *et al.*, 2004) and (C) the short *q*-mer frequency distributions (or *q-mer distributions* in short) of individual sufficiently long reads (Chatterji *et al.*, 2008; Prabhakara and Acharya, 2011; Teeling *et al.*, 2004; Wu and Ye, 2011; Yang *et al.*, 2010a,b) sampled from the same genome or similar genomes are similar (Yang *et al.*, 2010a,b).

AbundanceBin (Wu and Ye, 2011) groups reads based on Observation (A) but fails when the species in the sample have similar abundance. TOSS (Tanaseichuk *et al.*, 2011) bins reads based on Observations (A) and (B), and since TOSS relies on AbundanceBin to handle genomes with different abundances, it carries all the shortcomings of AbundanceBin. MetaCluster 4.0 (Wang *et al.*, 2012) has three phases: Phase 1 groups reads together based on Observation (B); Phase 2 derives the *q*-mer distribution of each group and Phase 3 merges the groups of reads based on Observation (C) by the well-known *K*-means clustering approach. MetaCluster 4.0 handles high-abundance species (with different abundances) in Phase 1 by having similar numbers of groups for each species (high-abundance species will have more reads in their groups). MetaCluster 4.0 works reasonably well for those species whose abundance (sequencing depth) of at least 10×, even in a sample with 100 species.

For easy discussion, we classify the abundances into three categories: (i) high abundance: at least 10×; (ii) low abundance: 6× to 10×; and (iii) extremely low abundance: at most 5×. There are at least two problems that MetaCluster 4.0 fails to address. (i) Interference from extremely low-abundance species: MetaCluster 4.0 does not perform well even for high-abundance species when there are many extremely low-abundance species in the sample. Table 1 shows such a case for a sample of 100 species with 85 extremely low-abundance species. MetaCluster 4.0 can only detect four high-abundance species. (ii) Difficulty in recovering low-abundance species: MetaCluster 4.0 does not work well for low-abundance species even without too much noise from extremely low-abundance species. Table 3 shows an example of 20 species in a sample with only five extremely low-abundance species, for which MetaCluster 4.0 is not able to bin any of the low-abundance species.

**Table 1.** Performance on Dataset A with 100 species (15 high abundance and 85 extremely low abundance)

	Species discovered		Sensitivity		Overall performance			
	≥ 10×	*<* 6×	≥ 10×	*<* 6×	Precision	Sensitivity	Memory	Time (min)
MetaCluster4.0	4	0	0.79	–	0.67	0.79	29G	70
MetaCluster5.0	14	0	0.90	–	0.92	0.90	20G	38

In fact, none of the existing binning tools can handle these two problems, which are common in real datasets. For example, in samples from real applications, there are usually many reads (can be *>*50% of the total reads) sampled from extremely low-abundance genomes and there is usually a portion of low-abundance species that may be significant to the biological system (e.g. Qin *et al.*, 2010). An ideal binning solution should be able to identify all species regardless of their abundance. However, it is very difficult to bin extremely low-abundance species and we leave it as an open problem to identify all these extremely low-abundance species. In this article, we aim at identifying low-abundance species, in addition to indentifying high-abundance species when there is interference from the extremely low-abundance species. We first discuss why the aforementioned two problems are difficult to solve.

### 1.1 Difficulties of the problems

Recall that existing tools are all based on the aforementioned three Observations. Note that Observation (C) relies very much on the grouping of reads using Observations (A) and (B). However, a direct application of Observations (A) and (B) cannot solve the problems satisfactorily. Binning based on Observation (A) has been known to fail if some species in the sample have similar abundance, a feature often found for real datasets. Even worse, the abundances of a large number of species in a real sample usually form a continuous spectrum from extremely low-abundance (1×) to moderately high-abundance (say 20×). This continuous spectrum of abundances causes reads from different species to mix together (we show an example of this mixing together in Section 3 using AbundanceBin).

In applying Observation (B), there is the technical issue of picking the value *w*. Intuitively, picking a larger *w* can decrease the number of false positives (reads from different species mixing together in a group), but also might make the groups too small for the application of Observation (C) and thus can only favor high-abundance species. Low-abundance species will likely be missed due to not enough coverage to connect the reads with large *w*-mers. Picking a smaller *w* can make the groups bigger and allow more low-abundance species to be identified but will increase the number of false positives, especially when there is noise from extremely low-abundance species. Since there are high-abundance species, reads from the low-abundance species will likely be merged into the groups of high-abundance species or be mixed together with some reads from extremely low-abundance species. Thus, how to set this *w* is not trivial and a single *w* value may not be appropriate for obtaining both high-abundance and low-abundance species.

On the other hand, while extremely low-abundance species may not have enough reads for binning, low-abundance species seem to have enough reads for binning if we can eliminate the noise from extremely low-abundance species and separate them from high-abundance species.

### 1.2 Our contributions

In this article, we introduce an unsupervised binning tool, MetaCluster 5.0, which extends MetaCluster 4.0 (Wang *et al.*, 2012) with the addition of a few techniques to handle the abovementioned problems. MetaCluster 5.0 works in a two-round manner. In the first round, we group reads from high-abundance species, and in the second round, we handle reads from low-abundance species.

- Filtering the extremely-low-abundance species. Since reads from extremely-low-abundance species have adverse effects on the binning results, MetaCluster 5.0 removes these reads at the initial step based on Observation (A) so as to improve the accuracy of the results and so as to reduce the size and thus the complexity of the problem. In order not to mistakenly remove reads (with some errors) from high-abundance species, we apply the following observation to remove the reads. If a read comes from an extremely low-abundance species, it is likely that all its *k*-mers’ frequencies (if *k* is large enough) are low and it should be removed, but note that the value of *k* is not the same as that of *w* in *w*-mers. *w*-mers aim at uniqueness in a genome while *k*-mers aim at handling sequencing errors. A read will not be removed as long as one of its *k*-mers has high frequency as some of its *k*-mer frequencies (which contain errors) for high-abundance species can be very low. For details on how to set *k* and the thresholds for filtering, please refer to Section 2.
- Low-abundance species grouping. After filtering extremely low-abundance species using *k*-mer frequencies and after grouping reads from high-abundance species with longer *w*-mers overlaps in the first round, we will have reads mostly from low-abundance species remaining in our dataset for the second round. However, a direct application of Observation (B) using a smaller *w* may still fail since there is still a chance that reads from different genomes are grouped, and any grouping mistake may affect the quality of the group significantly. So, we adopt a multiple *w* approach, we first use a large *w* to group reads with high confidence, then use a smaller *w* to increase the size of each group to facilitate *q*-mer distribution estimation [Observation (C)].

By removing reads from low-abundance species and by considering reads from different ranges of abundance ratio genomes one after another, MetaCluster 5.0 also enjoys the advantage of using less memory and running time.

We have compared MetaCluster 5.0 with existing binning method MetaCluster 4.0. We used three simulated datasets (one with the problem of many extremely low-abundance species and one with the problem of low-abundance species without too many extremely low-abundance species and one with both problems) and a real dataset [from (Qin *et al.*, 2010)] to evaluate the tools. MetaCluster 5.0 outperforms MetaCluster 4.0 substantially for all three simulated datasets. MetaCluster 5.0 is able to identify almost all low-abundance species in all cases with high sensitivity and precision, while MetaCluster 4.0 can only identify very few and sometimes none at all. In terms of high-abundance species, MetaCluster 5.0 also performs better than MetaCluster 4.0 especially in datasets with many extremely low-abundance species (with 15 species in total, MetaCluster 5.0 identifies 14 species while MetaCluster 4.0 can only identify 4) with even higher precision and sensitivity. For the real dataset, MetaCluster 5.0 can also identify all five known low-abundance species and six out of seven high-abundance species, while MetaCluster 4.0 cannot identify any of the low-abundance species and can only identify the three most abundant species.

## 2 METHODS

**Fig. 1.**
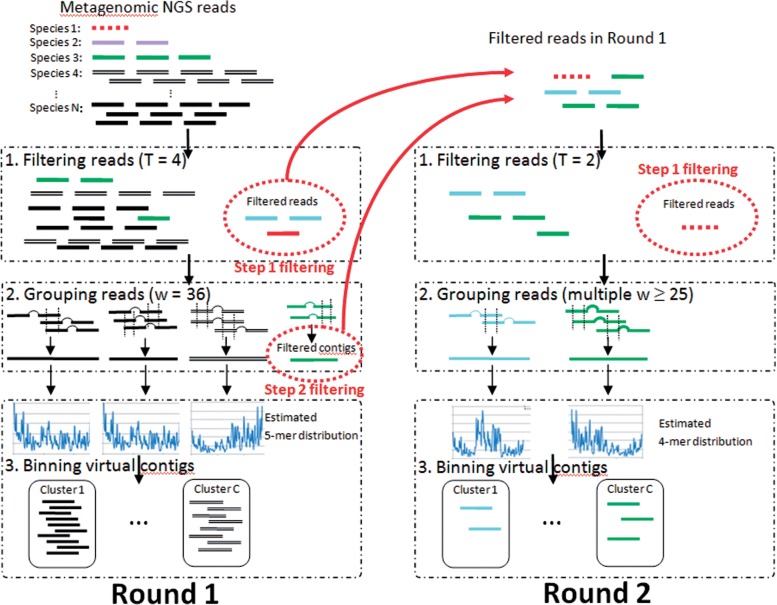
Workflow of MetaCluster 5.0

MetaCluster 5.0 is a two-round binning method using Observations (A), (B) and (C), developed based on MetaCluster 4.0 (Wang *et al.*, 2012). In the first round, it filters those reads sampled from both low-abundance and extremely low-abundance genomes and bins the reads sampled from high-abundance genomes (or species) only. In the second round, it filters those reads sampled from extremely low-abundance genomes and bins the reads sampled from low-abundance genomes. Since some reads are filtered in each round (up to 50% of reads), MetaCluster 5.0 requires less memory and running time (usually proportional to the square of the number of reads) than MetaCluster 4.0. A workflow of MetaCluster 5.0 is shown in Figure 1 and we will describe MetaCluster 5.0 in detail in the following sections.

### 2.1 First round

#### 2.1.1 Filtering reads

MetaCluster 5.0 first filters reads from low-abundance genomes as well as reads with many error bases. Based on the observation that *k*-mer frequency from reads of a genome is usually linearly proportional to that genome's abundance [Observation (A)], reads with all *k*-mers appearing rarely in the dataset are likely to be sampled from low-abundance genomes. MetaCluster 5.0 filters those reads with all *k*-mers appearing at most *T* times in the whole dataset (Step 1, filtering). We have a strict filtering requirement that all *k*-mers instead of part of them to be appearing at most *T* times because some reads sampled from high-abundance genomes may contain *k*-mers with low frequencies because of sequencing bias or sequencing errors. We pick *k* = 16 based on the research findings in (Fofanov *et al.*, 2004) and calculate the threshold *T* according to the target genome's abundance as follows.

Given a target abundance (sequencing depth) *d*, read length *r*, genome length *g*, sequencing error rate *e* and reads randomly picked from the genome, the expected number of sampled reads from this genome is

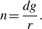


For a particular *k*-mer, the probability of an arbitrary sampled read contains this *k*-mer is

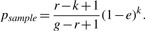


Assume that the frequency of this *k*-mer in the dataset follows normal distribution:

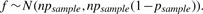


It is easy to see that frequency *f* is not sensitive to genome length *g* as *g* is much larger than read length *r*. Assume that we want to preserve those reads sampled from genome with abundance at least *d* with probability at least *p* in the first round. We can calculate the value of *T* such that:

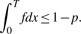

For example, when read length *r* = 75, *d* = 10, *p* = 80%, *k* = 16 and *e* = 0.01, g ~ 3 million, we should pick *T* = 4.

#### 2.1.2 Grouping reads

The reads are then grouped based on the common *w*-mer appearing in the reads [Observation (B)]. Two groups of reads are merged if and only if each group contains a read with common *w*-mers. In the first round, since the target abundance is 10× or more, we let *w* = 36 so as to have two reads sampled in a nearby region of a genome merged together in the same group with 99% accuracy according to the study by Wang *et al*. (2012). Some reads sampled from low-abundance genomes may still not be filtered in the first step. Since the probability of reads sampled in nearby region of a low-abundance genome are merged into the same group is low, the sizes of these groups will be small. As each group of reads represents a virtual contig of a genome, those small groups of reads whose virtual contigs are of length *<*1000 bp will be filtered (Step 2, filtering).

#### 2.1.3 Binning virtual contigs

The 5-mer distribution of each virtual contig is estimated and the virtual contigs are grouped using *K*-means clustering method based on the Spearman distance of the 5-mer distribution [Observation (C)]. Although this step is similar to MetaCluster 4.0, the length of virtual contigs produced by MetaCluster 5.0 is much longer than those produced by MetaCluster 4.0. Thus, we can estimate the 5-mer distribution, instead of 4-mer distribution used in MetaCluster 4.0, to get a better binning result.

### 2.2 Second round

After the first round, reads sampled from high-abundance genomes have been binned. In the second round, we target for binning reads sampled from low-abundance genomes. For filtering reads sampled from extremely low-abundance genomes (sequencing depth *<* 6×), we applied the Step 1 filtering again, but with a lower threshold *T*, say *T* = 2. In other words, a read will be discarded in this step if and only if all its *k*-mers are unique. As such reads will never be grouped in later phases, we filter them to save space and time.

#### 2.2.1 Grouping reads with multiple w

In order to guarantee that two reads sampled in a nearby region of a low-abundance genome (with sequencing depth 6× or more) can be merged in the same group with 99% probability, we should use a smaller *w* (*w* = 22 deduced from the study in (Wang *et al.*, 2012) for common *w*-mer grouping). However, reads sampled from different genomes may be merged incorrectly as *w* is small (false positives). For reducing the false-positive effect of small *w*, MetaCluster 5.0 groups the reads sharing longer *w*-mer first because two reads sharing a longer common substring have higher probability to be from the same species.

Since the abundance is low, some groups of reads may be small and may represent short virtual contigs. Thus, the 4-mer distribution, instead of 5-mer distribution used in the first round, is estimated based on each virtual contig for binning.

### 2.3 Time and space complexity

Since the numbers of *k*-mers and *w*-mers are at most *nr* where *n* is the number of input reads and *r* is the length of read. The space complexity is *O*(*nr*).

For the time complexity, *O*(*nr*) time is required for filtering reads, *O*[∑_I≤K_(*nrv_i_*^2^)] time is required for grouping reads with at most *nr* different *w*-mers and the frequency of the *i*-th *w*-mer is *v_i_*. The virtual contigs for *q*-mer distribution can be constructed in *O*(*nr*) times and the *K*-means clustering takes *O*[*gtc*·lg(*c*)] time where *g* is the number of groups of reads, *t* is the number of iterations and *c* is the initial number of centers used in the *K*-means algorithm. The total time complexity is *O*[*gtc*·lg(*c*)+ ∑ *_i≤K_* (*nrv_i_*^2^)].

## 3 RESULTS

We evaluate the performance of MetaCluster 5.0 on simulated data and real data. MetaCluster 4.0, AbundanceBin and TOSS. (The software tool of TOSS was obtained through a private communication with the authors of the article.) are the latest unsupervised binning tools for NGS reads. However, TOSS and AbundanceBin are very slow. TOSS (based on output of AbundanceBin) cannot finish any of the datasets in a week. AbundanceBin can only finish the smallest dataset with 8.3 million reads from 20 species, but the result is not satisfactory and all the reads grouped together even though the species are of uneven abundance. Since the performance of TOSS relies on AbundanceBin results, it is likely that TOSS may not perform well for species with similar abundance. As MetaCluster 4.0 outperforms AbundanceBin and TOSS in many situations (Wang *et al.*, 2012), we mainly compare the performances of MetaCluster 5.0 and MetaCluster 4.0.

All experiments were performed on a UNIX machine with 4CPU of Intel Xeon X5650@2.4GHz.

### 3.1 Experiments on simulated data

The simulated data are generated based on genomes in National Center for Biotechnology Information (NCBI) database (ftp.ncbi.nih.gov/genomes/) and the NCBI taxonomy is downloaded from ftp://ftp.ncbi.nih.gov/pub/taxonomy/. Given a set of genomes with the corresponding abundances, a set of length-75 paired-end reads are randomly generated with 1% sequencing error and 250 ± 50 bp insert distance from the genomes. The performances of the binning algorithms are evaluated on precision, sensitivity and the number of discovered species. Assume there are *N* genomes in the dataset and a binning algorithm outputs *M* clusters *C_i_*(1 ≤ *i* ≤ *M*). Let *R_ij_* be the number of reads in *C_i_* which are from genome *j* and *C_j_* represents genome *j*_0_ when *R_ij_*_0_ = max*_j_ R_ij_*. The overall precision and sensitivity is calculated as

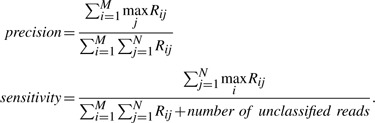


If *M >> N*, the majority of reads in each cluster probably belongs to a single genome and thus precision would be high. However, sensitivity would be low as some genomes are represented by multiple clusters. If *M << N*, some clusters would contain reads from multiple genomes and precision would be low. Thus, precision increases while sensitivity decreases with the number of predicted clusters.

Consider all the reads sampled from a particular species *S*, if there is a cluster *C* such that *>*50% of the reads are sampled from *S* and *>*50% reads sampled from *S* are in cluster *C*, we say species *S* is discovered by cluster *C*. Note that each species can be discovered by at most one cluster and each cluster can discover at most one species.

We simulated three datasets with different difficulties mentioned in the introduction: (i) many reads from extremely-low-abundance genomes, (ii) some reads from low-abundance genomes and (iii) reads from low-abundance genomes as well as many reads from extremely-low-abundance genomes.

#### 3.1.1 Noise from extremely low-abundance species

If there are many reads sampled from extremely-low-abundance genome, reads sampled from high-abundance genomes are difficult to be binned well. Existing binning algorithms cannot perform well on such datasets. To illustrate this, we construct a dataset (Dataset A) with 100 species randomly picked from 18 genera. Twenty of them are of sequencing depth 1×; 20 of them are of sequencing depth 2×; 20 of them are of sequencing depth 3×; 20 of them are of sequencing depth 4×; 5 of them are of sequencing depth 5×; the remaining 15 are of sequencing depth 11×, 12×, …, 25×. In total, there are 23.2 million reads. MetaCluster 4.0 and MetaCluster 5.0 were tested on this dataset and the performance is shown in Table 1.

Among the 15 high-abundance species (≥ 10×), MetaCluster 5.0 discovers 14 and MetaCluster 4.0 discovers four species. The poor performance of MetaCluster 4.0 is due to the noise introduced by the large number of reads from extremely low-abundance species. On the other hand, as MetaCluster 5.0 can successfully filter the reads from extremely-low-abundance species (*<*6×) as shown in Table 2, most of the high-abundance species can be discovered. The only species not discovered by MetaCluster 5.0 appears in three clusters and all these three clusters have high precision (*>*90% reads in these clusters are from the missing species).

**Table 2.** Percentage of filtered reads by MetaCluster 5.0 (Dataset A)

	First round			Second round
	≥ 10× (%)	*<* 6× (%)	≥ 6× (%)	*<* 6× (%)
Filter step 1	3.6	90.4	12	9
Filter step 2	6.2	95.4		–

Obviously, MetaCluster 5.0 can produce results with higher sensitivity and precision. Besides, as some of the reads can be filtered in each round, MetaCluser5.0 requires less space and time.

#### 3.1.2 Low-abundance species

MetaCluter 5.0 optimizes the two binning rounds to discover more species, while existing binning algorithms can only discover high-abundance species. To illustrate this, we constructed a dataset (Dataset B) with 20 species randomly picked from four genera, with sequencing depths are 1×, 2×, 3×, …, 20× and 8.3 million reads in total.

The binning performance of MetaCluster 4.0 and MetaCluster 5.0 is shown in Table 3. While MetaCluster 5.0 can successfully discover all 11 high-abundance species, MetaCluster 4.0 can discover nine of them. Moreover, MetaCluster 5.0 can discover three out of four low-abundance species and MetaCluster 4.0 can discover none of them. Besides, the precision and sensitivities of MetaCluster 5.0 are higher than those of MetaCluster 4.0 in all categories. It is because MetaCluster 5.0 can filter the reads from low-abundance species in the first round (Table 4) and bin them successfully in the second round. However, the sensitivity of low-abundance species is a bit low because of their short virtual contigs due to the low coverage. Thus, the binning performance in second round is not as good as the first round.

**Table 3.** Performance on Dataset B with 20 species (11 high abundance, four low abundance and five extremely low abundance)

	Species discovered			Sensitivity		Overall performance			
	≥ 10×	(6×, 10×)	*<* 6×	≥ 10×	(6×, 10×)	Precision	Sensitivity	Memory	Time (min)
MetaCluster4.0	9	0	0	0.79	–	0.82	0.82	12.5G	16
MetaCluster5.0	11	3	0	0.87	0.80	0.92	0.87	7.7G	14

**Table 4.** Percentage of filtered reads by MetaCluster 5.0 (Dataset B)

	First round			Second round	
	≥ 10× (%)	[6×, 10×] (%)	*<* 6× (%)	≥ 6× (%)	*<* 6× (%)
Filter step 1	3.4	37.1	86.2	20	22
Filter step 2	6.2	61.4	97.5	–	

#### 3.1.3 Dataset with both difficulties

The above two datasets demonstrate that MetaCluster 5.0 can solve the two difficulties mentioned in Section 1 independently. Here, we construct a dataset (dataset C) which has both difficulties. One-hundred species are randomly picked from 18 genera; 20 of them are of sequencing depth 1×; 20 of them are of sequencing depth 2×; 20 of them are of sequencing depth 3×; 20 of them are of sequencing depth 4×; 20 of them are of sequencing depth 6×, 7×,…, 25×, respectively. In total, there are 24.3 million reads.

The performance is shown in Tables 5 and 6. Among all the 16 high-abundance species (≥ 10×), MetaCluster 5.0 discovers all of them while MetaCluster 4.0 only discovers nine of them. For the four low-abundance species (≥ 6× and *<*10×), MetaCluster 5.0 discovers three of them while MetaCluster 4.0 only discovers one of them. MetaCluster 5.0 has much higher precision (0.87 versus 0.62); better sensitivity (0.88 versus 0.80) and runs faster than MetaCluster 4.0.

**Table 5.** Performance on Dataset C with 100 species (16 high abundance, four low abundance and 80 extremely low abundance)

	Species discovered			Sensitivity		Overall performance			
	≥ 10×	[6×, 10×)	*<* 6×	≥ 10×	[6×, 10×]	Precision	Sensitivity	Memory	Time (min)
MetaCluster4.0	9	1	1	0.81	0.60	0.62	0.80	31G	87
MetaCluster5.0	16	3	3	0.91	0.72	0.87	0.88	21G	45

**Table 6.** Percentage of filtered reads by MetaCluster 5.0 (Dataset C)

	First round			Second round	
	≥ 10× (%)	[6×, 10×] (%)	*<* 6× (%)	≥ 6× (%)	*<* 6× (%)
Filter step 1	3.5	39.9	93	13	11
Filter step 2	4.6	60.1	97	–	

### 3.2 Experiments on real data

To evaluate the performance of MetaCluster 5.0 on real dataset, the dataset provided by the study of Qin *et al.* in (Qin *et al.*, 2010), which collected samples from the feces of 124 European adults, is studied. Since the dataset contain different samples with read length varies from 44 to 75 bp, we selected one sample from 57 Denmark adults with 75-bp pair-end reads with matches with the common experimental setting.

Since there are many reads sampled from genomes with unknown reference, it is difficult evaluate the performance of a binning algorithm. In order to calculate the precision and sensitivity of the binning algorithms, all reads are not sampled from the most abundant 15 species with known reference genomes are filtered. Software BLAT (Kent, 2002) to map reads to the 15 reference genomes with 5% mismatch allowed. After filtering, there are 8 million reads in the dataset. The results obtained by MetaCluster 5.0 are shown in Table 7.

**Table 7.** Performance of MetaCluster 5.0 on the real dataset

Groups	Major species	Precision	Sensitivity
Group 1	*Bacteroides uniformis*	0.82	0.84
Group 2	*Alistipes putredinis*	0.79	0.54
Group 3	*Parabacteroides merdae*	0.65	0.65
Group 4	*Eubacterium hallii DSM 3355*	0.98	0.70
Group 5	*Ruminococcus torques L2 14*	0.59	0.55
Group 6	*Faecalibacterium*	0.76	0.69
Group 7	*Dorea formicigenerans ATCC 27755*	0.59	0.78
Group 8	*Roseburia intestinalis M501*	0.71	0.62

In this dataset, there are three low-abundance species (between 6× and 10×), and MetaCluster 5.0 can discover all of them. For the six high-abundance species (≥ 10×), MetaCluster 5.0 finds five of them. The only missing one is of sequencing depth 11× and mixed with other species from the same genus. As highly related genomes share too many common regions, their reads can be easily mixed together. MetaCluster 5.0 has an overall precision and sensitivity of *>*70%.

MetaCluster 4.0 cannot discover any of the low-abundance species and it can only discover the most abundant three species in the dataset. As MetaCluster 4.0 outputs too many clusters, we list the discovered species in Table 8. Its overall precision and sensitivity are both lower than those of MetaCluster 5.0.

**Table 8.** Performance of MetaCluster 4.0 on the real dataset

Groups	Major species	Precision	Sensitivity
Group 1	*Bacteroides uniformis*	0.79	0.53
Group 2	*Alistipes putredinis*	0.77	0.56
Group 3	*Roseburia intestinalis M501*	0.51	0.89

## 4 CONCLUSION

Metagenomics binning remains a crucial step in metagenomics analysis. Existing unsupervised binning algorithms fail to bin reads from low-abundance species or cannot bin reads from high-abundance species when there are many reads from extremely low-abundance species. In this article, we introduce MetaCluster 5.0 that overcomes these two problems by binning the reads in two rounds with a filtering step to remove noise from extremely low-abundance species. MetaCluster 5.0 outperforms existing binning algorithms for both simulated and real biological datasets.

A trivial extension of MetaCluster 5.0 is to bin reads with different abundances using multiple (more than two) rounds. However, the filtering error may accumulate in each round and fewer reads can be preserved in each subsequent round. One possible direction is to reuse some of the reads used in the previous rounds. How to make the multiple-round approach more effective requires more in-depth investigation. Another important direction for future work is to bin reads from extremely low-abundance species, which is basically a well-known open problem in this area.

*Funding*: This research is partially supported by HK GRF grant [HKU 711112, HKU 719611E].

*Conflict of Interest:* none declared.
